# Novel applications of Convolutional Neural Networks in the age of Transformers

**DOI:** 10.1038/s41598-024-60709-z

**Published:** 2024-05-01

**Authors:** Tansel Ersavas, Martin A. Smith, John S. Mattick

**Affiliations:** 1 School of Biotechnology and Biomolecular Sciences, UNSW Sydney, Sydney, NSW 2052 Australia; 2 Department of Biochemistry and Molecular Medicine, Faculty of Medicine, Université de Montréal, Montréal, QC H3C 3J7 Canada; 3 CHU Sainte-Justine Research Centre, Montreal, Canada; 4 UNSW RNA Institute, UNSW Sydney, Australia

**Keywords:** Computational science, Machine learning

## Abstract

Convolutional Neural Networks (CNNs) have been central to the Deep Learning revolution and played a key role in initiating the new age of Artificial Intelligence. However, in recent years newer architectures such as *Transformers* have dominated both research and practical applications. While CNNs still play critical roles in many of the newer developments such as Generative AI, they are far from being thoroughly understood and utilised to their full potential. Here we show that CNNs can recognise patterns in images with scattered pixels and can be used to analyse complex datasets by transforming them into pseudo images with minimal processing for any high dimensional dataset, representing a more general approach to the application of CNNs to datasets such as in molecular biology, text, and speech. We introduce a pipeline called *DeepMapper*, which allows analysis of very high dimensional datasets without intermediate filtering and dimension reduction, thus preserving the full texture of the data, enabling detection of small variations normally deemed ‘noise’. We demonstrate that *DeepMapper* can identify very small perturbations in large datasets with mostly random variables, and that it is superior in speed and on par in accuracy to prior work in processing large datasets with large numbers of features.

## Introduction

There are exponential increases in data^1^ especially from highly complex systems, whose non-linear interactions and relationships are not well understood, and which can display major or unexpected changes in response to small perturbations, known as the ‘Butterfly effect’^2^.

In domains characterised by high-dimensional data, traditional statistical methods and Machine Learning (ML) techniques make heavy use of feature engineering that incorporates extensive filtering, selection of highly variable parameters, and dimension reduction techniques such as Principal Component Analysis (PCA)^3^. Most current tools filter out smaller changes in data, mostly considered artefacts or `noise`, which may contain information that is paramount to understanding the nature and behaviour of such highly complex systems^4^.

The emergence of Deep Learning (DL) offers a paradigm shift. DL algorithms, underpinned by adaptive learning mechanisms, can discern both linear and non-linear data intricacies, and open avenues to analyse data that is not possible or practical by conventional techniques^5^, particularly in complex domains such as image, temporal sequence analysis, molecular biology, and astronomy^6^. DL models, such as *Convolutional Neural Networks* (CNNs)^7^, *Recurrent Neural Networks* (RNNs)^8^, *Generative Network*s^9^ and *Transformers*^10^, have demonstrated exceptional performance in various domains, such as image and speech recognition, natural language processing, and game playing^6^. CNNs and LSTMs were found to be great tools to predict behaviour of so called `chaotic` systems^11^. Modern DL systems often surpass human-level performance, and challenge humans even in creative endeavours.

CNNs utilise a unique architecture that comprises several layers, including convolutional layers, pooling layers, and fully connected layers, to process and transform the input data hierarchically^5^. CNNs have no knowledge of sequence, and therefore are generally not used in analysing time-series or similar data, which is traditionally attempted with Recurrent Neural Networks (RNNs)^12^ and Long Short-Term Memory networks (LSTMs)^8^ due to their ability to capture temporal patterns. Where CNNs have been employed for sequence or time-series analysis, 1-dimensional (1D) CNNs have been selected because of their vector based 1D input structure^13^. However, attempts to analyse such data in 1D CNNs do not always give superior results^14^. In addition, GPU (Graphical Processing Units) systems are not always optimised for processing 1D CNNs, therefore even though 1D CNNs have fewer parameters than 2-dimensional (2D) CNNs, 2D CNNs can outperform 1D CNNs^15^.

*Transformers*, introduced by Vaswani et al.^10^, have recently come to prominence, particularly for tasks where data are in the form of time series or sequences, in domains ranging from language modelling to stock market prediction^16^. *Transformers* leverage self-attention, a key component that allows a model to weigh and focus on various parts of an input sequence when producing an output, enabling the capture of long-range dependencies in data. Unlike CNNs, which use local receptive fields, self-attention weighs the significance of various parts of the input data^17^.

Following success with sequence-based tasks, *Transformers* are being extended to image processing. *Vision-Transformers* in object detection^18^, *Detection Transformers*^19^ and lately *Real-time Detection Transformers* all claim superiority over CNNs^20^. However, their inference operations demand far more resources than CNNs and trail CNNs in flexibility. They also suffer similar augmentation problems as CNNs. More recently, *Retentive-Networks* have been offered as an alternative to *Transformers*^21^ and may soon challenge the *Transformer* architecture.

### CNNs can recognise dispersed patterns

Even though CNNs are widely used, there are some misconceptions, notably that CNNs are largely limited to image data, and require established spatial relationships between pixels in images, both of which are open to challenge. The latter is of particular importance when considering the potential of CNNs to analyse complex non-image datasets, whose data structures are arbitrary.

Moreover, while CNNs are universal function approximators^22^, they may not always generalise^23^, especially if they are trained on data that is insufficient to cover the solution space^24^. It is also known that they can spontaneously generalise even when supplied with a small number of samples during training after overfitting, called ‘grokking’^25,26^. CNNs can generalise from scattered data if given enough samples, or if they grok, and this can be determined by observing changes to training versus testing accuracy and loss.

### Non-image processing with CNNs

While CNNs have achieved remarkable success in computer vision applications, such as image classification and object detection^7,27^, they have also been employed in other domains to a lesser degree with impressive results, including: (1) natural language processing, text classification, sentiment analysis and named entity recognition, by treating text data as a one-dimensional image with characters represented as pixels^16,28^; (2) audio processing, such as speech recognition, speaker identification and audio event detection, by applying convolutions over time frequency representations of audio signals^29^; (3) time series analysis, such as financial market prediction, human activity recognition and medical signal analysis, using one-dimensional convolutions to capture local temporal patterns and learn features from time series data^30^; and (4) biopolymer (e.g., DNA) sequencing, using 2D CNNs to accurately classify molecular barcodes in raw signals from Oxford Nanopore sequencers using a transformation to turn a 1D signal into 2D images—improving barcode identification recovery from 38 to over 85%^31^.

Indeed, CNNs are not perfect tools for image processing as they do not develop semantic understanding of images even though they can be trained to do semantic segmentation^32^. They cannot easily recognise negative images when trained with positive images^33^. CNNs are also sensitive to the orientation and scale of objects and must rely on augmentation of image datasets, often involving hundreds of variations of the same image^34^. There are no such changes in the perspective and orientation of data converted into flat 2D images.

In the realm of complex domains that generate huge amounts of data, augmentation is usually not required for non-image datasets, as the datasets will be rich enough. Moreover, introducing arbitrary augmentation does not always improve accuracy; indeed, introducing hand-tailored augmentation may hinder analysis^35^. If augmentation is required, it can be introduced in a data-oriented form, but even when using automated augmentation such as *AutoAugment*^35^ or *FasterAutoAugment*^36^, many of the augmentations (such as shearing, translation, rotation, inversion, etc.) should not be used, and the result should be tested carefully, as augmentation may introduce artefacts.

A frequent problem with handling non-image datasets with many variables is noise. Many algorithms have been developed for noise elimination, most of which are domain specific. CNNs can be trained to use the whole input space with minimal filtering and no dimension reduction, and can find useful information in what might be ascribed as ‘noise’^4,37^. Indeed, a key reason to retain ‘noise’ is to allow discovery of small perturbations that cannot be detected by other methods^11^.

### Conversion of non-image data to artificial images for CNN processing

Transforming sequence data to images without resorting to dimension reduction or filtering offers a potent toolset for discerning complex patterns in time series and sequence data, which potentiates the two major advantages of CNNs compared to RNNs, LSTMs and *Transformers*. First, CNNs do not depend on past data to recognise current patterns, which increases sensitivity to detect patterns that appear in the beginning of time-series or sequence data. Second, 2D CNNs are better optimised for GPUs and highly parallelizable, and are consequently faster than other current architectures, which accelerates training and inference, while reducing resource and energy consumption during in all phases including image transformation, training, and inference significantly.

**Figure 1:**
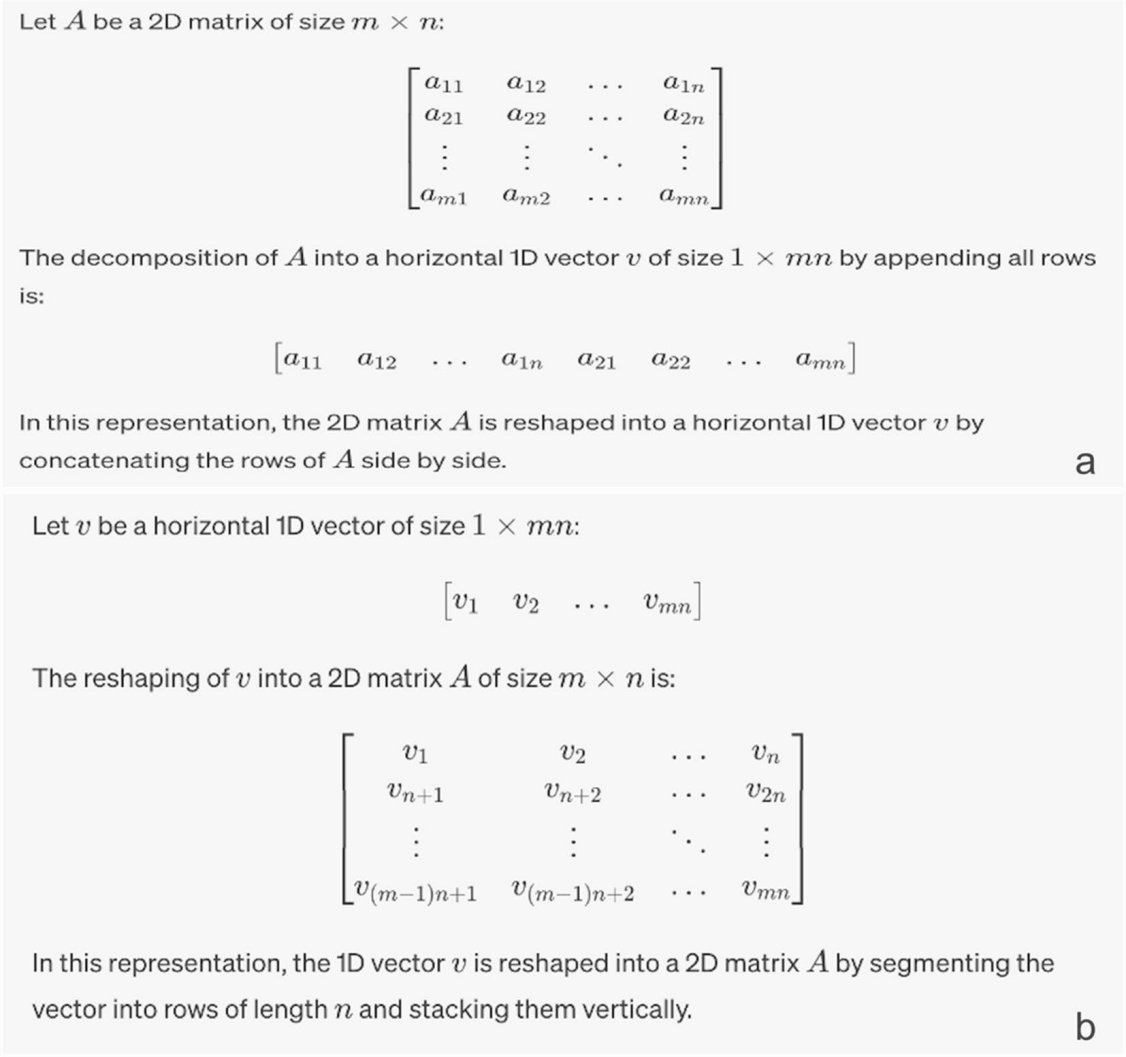
Conversion of images to vectors and vice versa. (**a**) Basic operation of transformation of an image to a vector, forming a sequence representation of the numeric values of pixels. (**b**) Transforming a vector to a matrix, forming an image by encoding numerical values as pixels. During this operation if the vector size cannot be mapped to m_X_n because vector size is smaller than the nearest m_X_n, then it is padded with zeroes to the nearest m_X_n.

Image data such as MNIST represented in a matrix can be classified by basic deep networks such as *Multi-level Perceptrons* (MLP) by turning their matrix representation to vectors (Fig. 1a). Using this approach analysis of images becomes increasingly complex as the image size grows, increasing the input parameters of MLP and the computational cost exponentially. On the other hand, 2D CNNs can handle the original matrix much faster than MLP with equal or better accuracy and scale to much larger images.

Just like how a simple neural network analyses a 2D image by turning it into a vector, the reciprocal is also true—data in a vector can be converted to a 2D matrix (Fig. 1b). Vectors converted to such matrices form arbitrary patterns that are incomprehensible to human eye. A similar technique for such mapping has also been proposed by Kovelarchuk et al. using another algorithm called CPC-R^38^.

### Attribution

An important aspect of any analysis is to be able to identify those variables that are most important and the degree to which they contribute to a given classification. Identifying these variables is particularly challenging in CNNs due to their complex hierarchical architecture, and many non-linear transformations^39^. To address this problem many ‘attribution methods’ have been developed to try to quantify the contribution of each variable (e.g., pixels in images) to the final output for deep neural networks and CNNs^40^.

Saliency maps serve as an intuitive attribution and visualisation tool for CNNs, spotlighting regions in input data that significantly influence the model's predictions^27^. By offering a heatmap representation, these maps illuminate key features that the model deems crucial, thus aiding in demystifying the model's decision-making process. For instance, when analysing an image of a cat, the saliency map would emphasise the cat's distinct features over the background. While their simplicity facilitates understanding even for those less acquainted with deep learning, saliency maps do face challenges, particularly their sensitivity to noise and occasional misalignment with human intuition^41–43^. Nonetheless, they remain a pivotal tool in enhancing model transparency and bridging the interpretability gap between ML models and human comprehension.

Several methods have been proposed for attribution, including *Guided Backpropagation*^44^, *Layer-wise Relevance Propagation*^45^, *Gradient-weighted Class Activation Mapping*^46^, *Integrated Gradients*^47^, *DeepLIFT*^48^, and *SHAP* (SHapley Additive exPlanations)^49^. Many of these methods were developed because it is challenging to identify important input features when there are different images with the same label (e.g., ‘bird’ with many species) presented at different scales, colours, and perspectives. In contrast, most non-image data does not have such variations, as each pixel corresponds to the same feature. For this reason, choosing attributions with minimal processing is sufficient to identify the salient input variables that have the maximal impact on classification.

### DeepMapper

Here we introduce a new analytical pipeline, *DeepMapper*, which applies a non-indexed or indexed mapping to the data representing each data point with one pixel, enabling the classification or clustering of data using 2D CNNs. This simple direct mapping has been tried by others but has not been tested with datasets with sufficiently large amounts of data in various conditions. We use raw data with minimal filtering and no dimension reduction to preserve small perturbations in data that are normally removed, in order to assess their impact.

The pipeline includes conversion of data, separation to training and validation, assessment of training quality, attribution, and accumulation of results in a pipeline. The pipeline is run multiple times until a consensus is reached. The significant variables can then be identified using attribution and exported appropriately.

**Figure 2:**
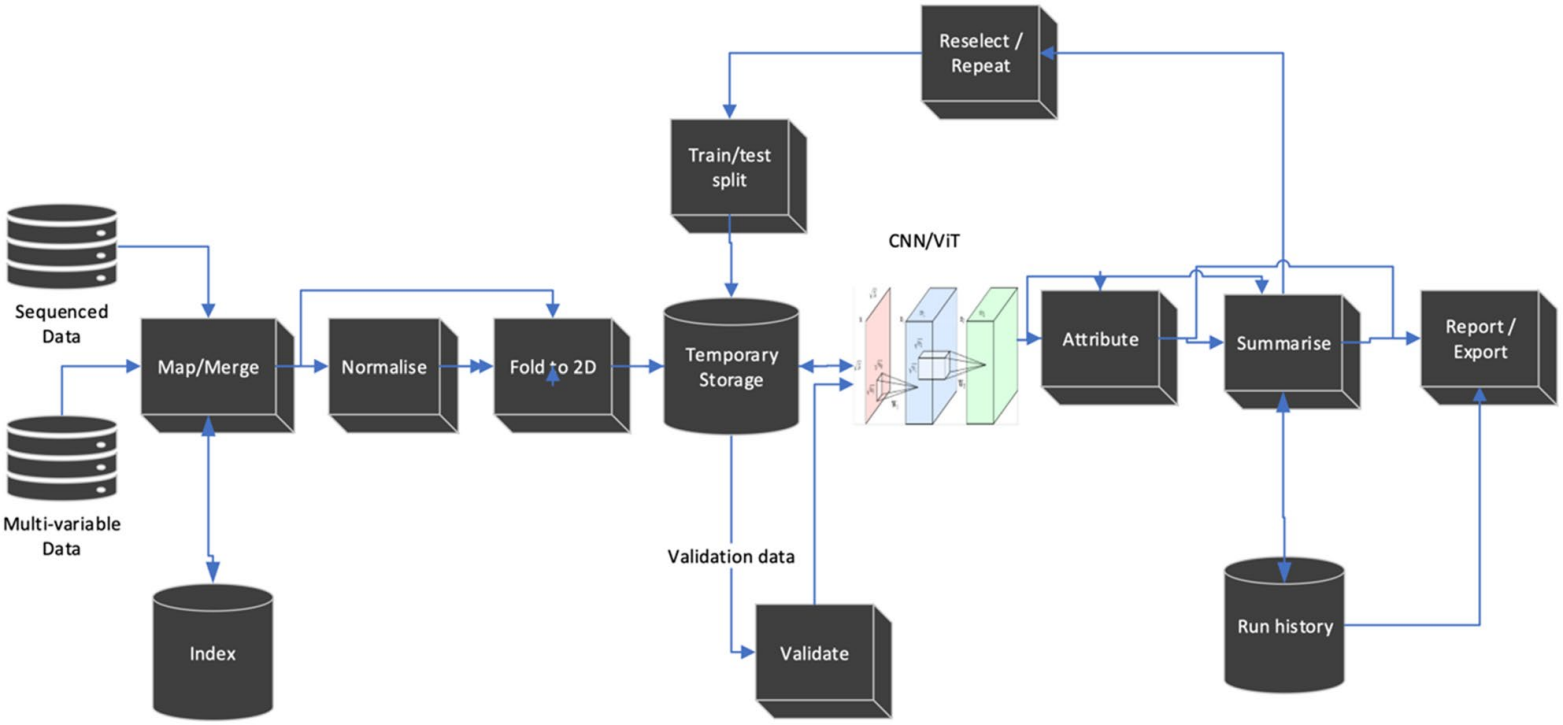
*DeepMapper* architecture. *DeepMapper* uses sequence or multi-variate data as input. The first step of *DeepMapper* is to merge and if required index input files to prepare them into matrix format. The data are normalised using log normalisation, then folded to a matrix. Folding is performed either directly with the natural order of the data or by using the index that is generated or supplied during the data import. After folding, the data are kept in temporary storage and separated to ‘train’ and ‘test’ using SciPy train test split. Training is done using either using CNNs that are supplied by the *PyTorch* libraries, or a custom CNN supplied (*ResNet18* is used by default). Intermediary results are run through attribution algorithms supplied by the *Captum*^51^ and saved to run history log. The run is then repeated until convergence is achieved, or until a pre-determined number of iterations are performed by shuffling training testing and validation data. Results are summarised in a report with exportable tables and graphics. Attribution is applied to true positives and true negatives, and these are translated back to features to be added to reports. Further details can be directly found in the accompanying code^50^.

The *DeepMapper* architecture is shown in Fig. 2. The complete algorithm of *DeepMapper* is detailed in the “Methods” section and the Python source code is supplied at GitHub^50^.

## Methods

*DeepMapper* is developed to implement an approach to process high-dimensional data without resorting to excessive filtering and dimension reduction techniques that eliminate smaller perturbations in data to be able to identify those differences that would otherwise be filtered out. The following algorithm is used to achieve this result:

1. Read and setup the running parameters.
2. Read the data into a tabulated form in the form of observations, features, and outcome (in the form of labels, or if self-supervised, the input itself).
  If the input data includes categorical features, these features should be converted to numbers and normalised before feeding to *DeepMapper*.
3. Identify features and labels.
4. Do only basic filtering that eliminates observations or features if all of them are 0 or empty.
5. Normalise features.
6. Transform tabulated data to 2-dimensional matrices as illustrated in Fig. 1a by applying a vector to matrix transformation.
7. If the analysis is supervised, then transform class labels to output matrices.
8. Begin iteration:
  - Separate the data into training and validation groups.
  - Train on the dataset for required number of epochs, until reaching satisfactory testing accuracy and loss, or maximum a pre-determined number of iterations.
  - If satisfactory testing results are obtained, then:
    i. Perform attributions by associating each result to contributing input pixels using Captum, a Python library for attributions^51^.
    ii. Accumulate attribution results by collecting the attribution results for each class.
  - If training is satisfactory:
    i. Tabulate attribution results by averaging accumulated attributions.
    ii. Save the model.
9. Report results.

The results of *DeepMapper* analysis can be used in 2 ways:

1. Supervised: *DeepMapper* produces a list of features that played a prominent role in the differentiation of classes.
2. Self-supervised: Highlights the most important features in differentiating observations from each other in a non-linear fashion. The output can be used as an alternative feature selection tool for dimension reduction.

In both modes, any hidden layer can be examined as latent space. A special bottleneck layer can be introduced to reduce dimensions for clustering purposes.

## Results

**Figure 3:**
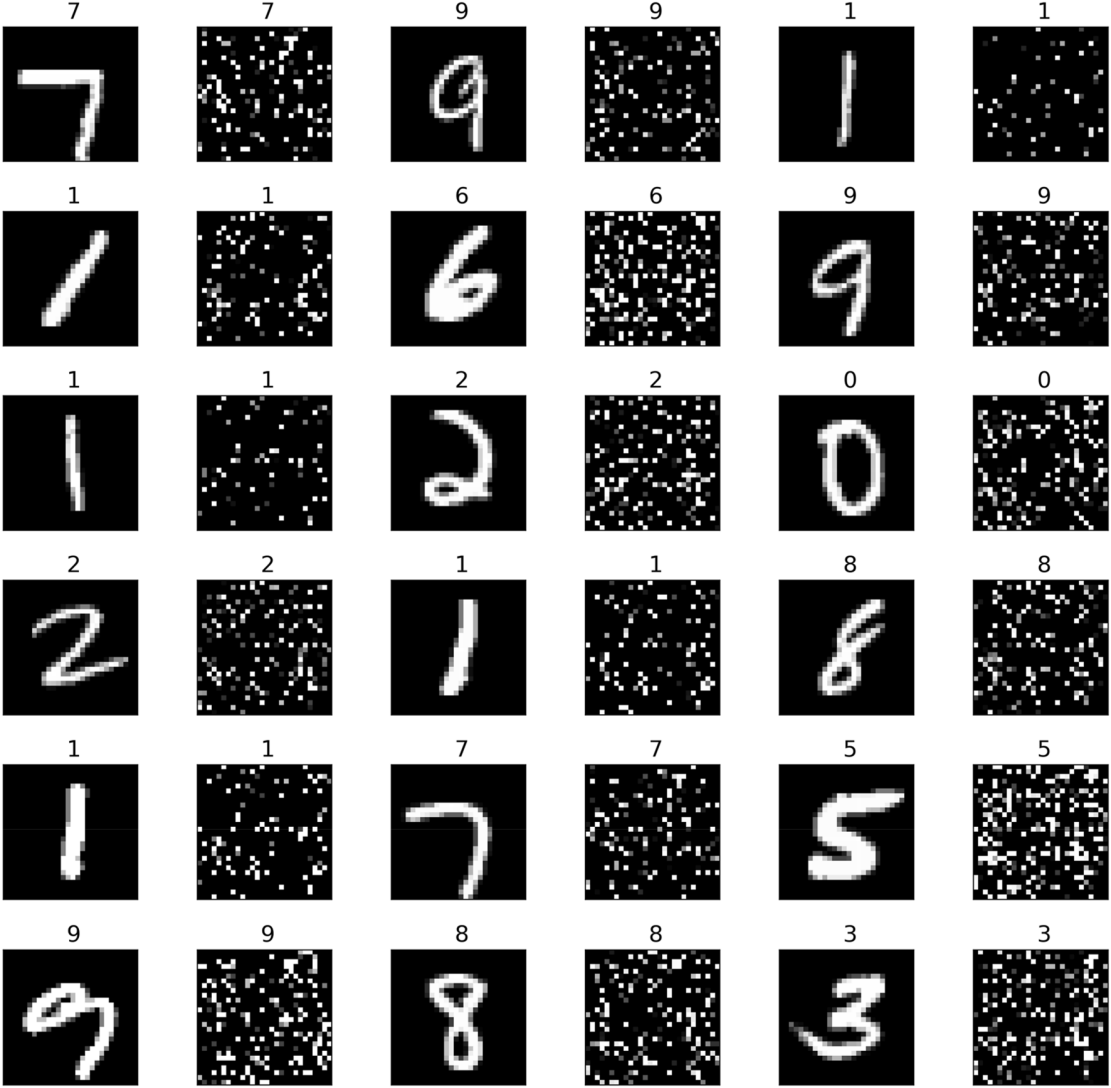
A sample from MNIST dataset (left side of each image) and its shuffled counterpart (right side).

We present a simple example to demonstrate that CNNs can readily interpret data with a well dispersed pattern of pixels, using the MNIST dataset, which is widely used for hand-written image recognition and which humans as well as CNNs can easily recognise and classify based on the obvious spatial relationships between pixels (Fig. 3). This dataset is a more complicated problem than datasets such as the *Gisette* dataset^52^ that was developed to distinguish between 4 and 9. It includes all digits and uses a full randomisation of pixels, and can be regenerated with the script supplied^50^ and changing the seed will generate different patterns.

**Figure 4:**
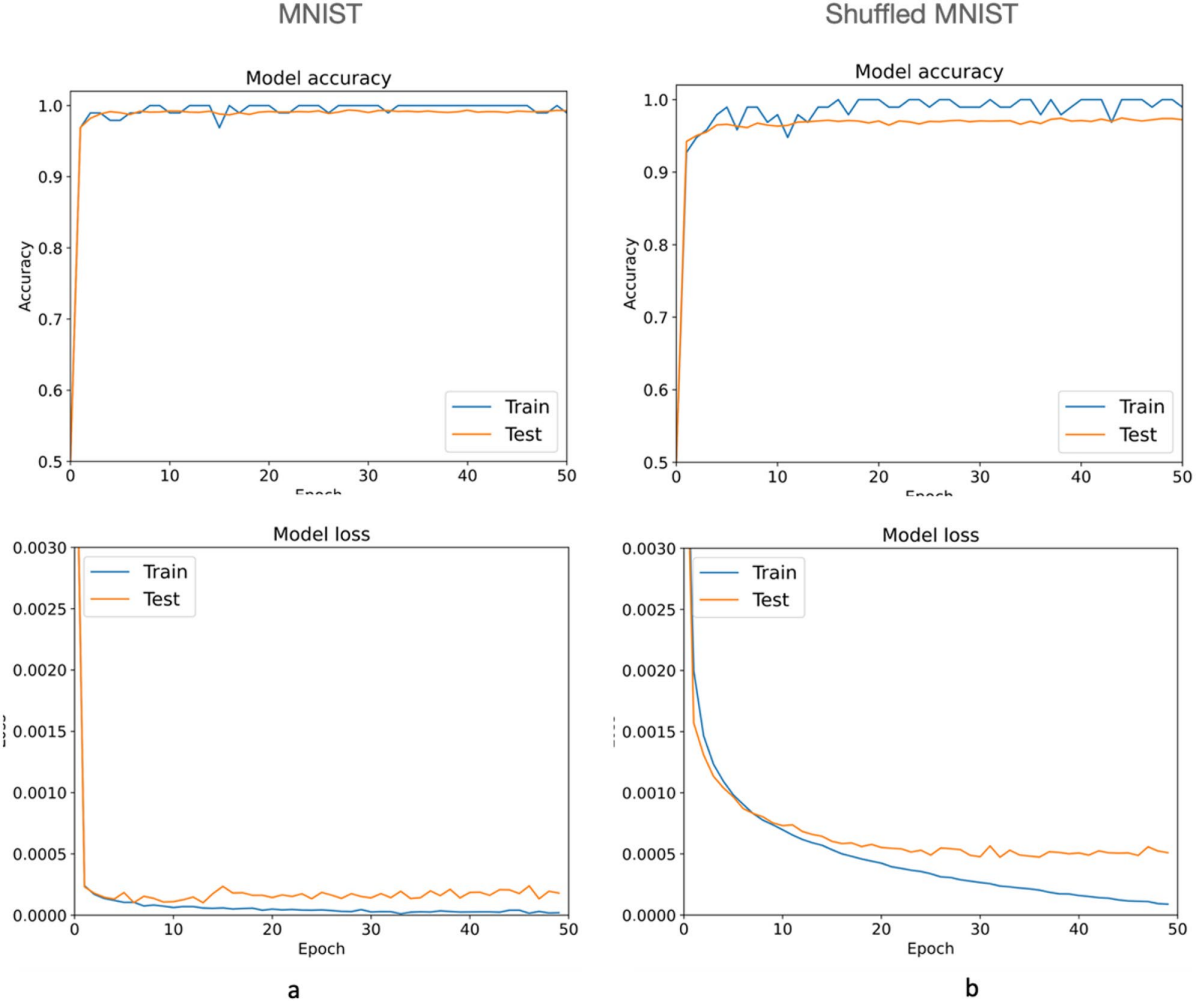
Results of training MNIST dataset (**a**) and the shuffled dataset (**b**) with PyTorch model *ResNet18*^50^. The charts demonstrate although the training continued for 50 epochs, about 15 epochs for shuffled images (**b**) would be enough, as further training starts causing overfitting. The decrease of accuracy between normal and shuffled images is about 3%, and this difference cannot be improved by using more sophisticated CNNs with more layers, meaning shuffling images cause a measurable loss of information, yet still hold patterns recognisable by CNNs.

We randomly shuffled the data in Fig. 3 using the same seed^50^ to obtain 60,000 training images such as those shown on the right side of each digit, and validated the results with a separate batch of 20,000 images (Fig. 3). Although the resulting images are no longer recognizable by eye, a CNN has no difficulty distinguishing and classifying each pattern with ~ 2% testing error compared to the reference data (Fig. 4). This result demonstrates that CNNs can accurately recognise global patterns in images without reliance on local relationships between neighbouring pixels. It also confirms the finding that shuffling images only marginally increases training loss^23^ and extends it to testing loss (Fig. 4).

### Testing DeepMapper

Finding slight changes in very few variables in otherwise seemingly random datasets with large numbers of variables is like finding a needle in a haystack. Such differences in data are almost impossible to detect using traditional analysis tools because small variations are usually filtered out before analysis.

We devised a simple test case to determine if *DeepMapper* can detect one or more variables with small but distinct variations in otherwise randomly generated data. We generated a dataset with 10,000 data items with 18,225 numeric variables as an example of a high-dimensional dataset using PyTorch’s uniform random algorithms^53^. The algorithm sets 18,223 of these variables to random numbers in the range of 0–1, and two of the variables into two distinct groups as seen in Table 1.

**Table 1:** Generated variables and their random ranges.

	Variable 20	Variable 17,998	Other variables
Class 0 range:	0.00–0.20	0.10–0.20	0.00–1.00
Class 1 range:	0.20–0.40	0.25–0.35	0.00–1.00

We call this type of dataset ‘Needle in a haystack’ (NIHS) dataset, where very small amounts of data with small variance is hidden among a set of random variables that is order(s) of magnitude greater than the meaningful components. We provide a script that can generate this and similar datasets among the source supplied^50^.

**Figure 5:**
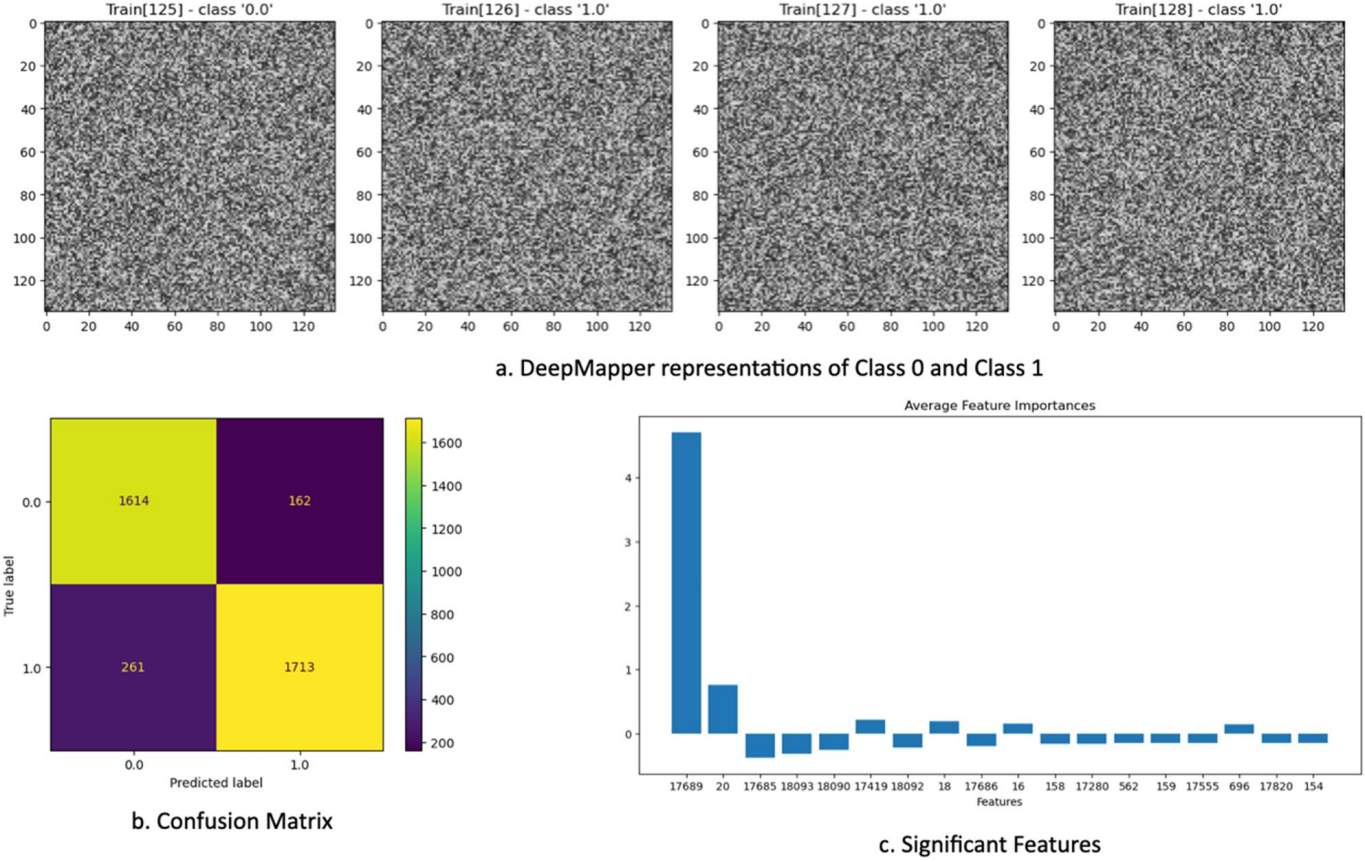
In this demonstration of analysis of high dimensional data with very small perturbations, *DeepMapper* can find these small variations in a few (in this example two) variables out of very large number of random variables (here 18,225). (**a**) *DeepMapper* representations of each record. (**b**) The result of the test run of the classification with unseen data (3750 elements). (**c**) The first and second variables in the graph are measurably higher than the other variables.

*DeepMapper* was able to accurately classify the two datasets (Fig. 5). Furthermore, using attribution *DeepMapper* was also able to determine the two datapoints that have different variances in the two classes. Note that *DeepMapper* may not always find all the changes in the first attempt as neural network initialisation of weights is a stochastic process. However, *DeepMapper o*vercomes this matter via multiple iterations to establish acceptable training and testing accuracies as described in the Methods.

### Comparison of DeepMapper with DeepInsight

*DeepInsight*^54^ is the most general approach published to date for converting non-image data into image-like structures, with the claim that these processed structures allow CNNs to capture complex patterns and features in the data. *DeepInsight* offers an algorithm to create images that have similar features collated into a “well organised image form”, or by applying one of several dimensionality reduction algorithms (e.g., t-SNE, PCA or KPCA)^54^. However, these algorithms add computational complexity, potentially eliminate valuable information, limit the abilities of CNNs to find small perturbations, and make it more difficult to use attribution to determine most notable features impacting analysis as multiple features may overlap in the transformed image. In contrast *DeepMapper* uses a direct mapping mechanism where each feature corresponds to one pixel.

To identify important input variables, *DeepInsight* authors later developed *DeepFeature*^55^ using an elaborate mechanism to associate image areas identified by attribution methods to the input variables. *DeepMapper* uses a simpler approach as each pixel corresponds to only one variable and can use any of the attribution methods to link results to its input space. While both *DeepMapper* and *DeepInsight* follow the general idea that non-image data can be processed with 2D CNNs, *DeepMapper* uses a much simpler and faster algorithm, while *DeepInsight* chooses a sophisticated set of algorithms to convert non-image data to images, dramatically increasing computational cost. The *DeepInsight* conversion process is not designed to utilise GPUs so cannot be accelerated by better hardware, and the obtained images may be larger than the number of data points, also impacting performance.

One of the biggest differences between *DeepFeature* and *DeepMapper* is that *DeepFeature* in many cases selects multiple features during attribution because *DeepInsight* pixels represent multiple values, whereas each *DeepMapper* pixel represents one input feature, therefore it can determine differentiating features with pinpoint accuracy at a resolution of 1 pixel per feature.

The *DeepInsight* manuscript offers various examples of data to demonstrate its abilities. However, many of the examples use low dimensions (20–4000 features) while today’s complex datasets may regularly require tens of thousands to millions of features such as in genome analysis in biology and radio-telescope analysis in astronomy. As such, several examples provided by *DeepInsight* have insufficient dimensions for a sophisticated mechanism such as *DeepMapper*, which should ideally have 10,000 or more dimensions as required by modern complex datasets. *DeepInsight* examples include a speech dataset from the TIMIT corpus with 39 dimensions, *Relathe* (text) dataset, which is derived from newsgroup documents and partitioned evenly across different newsgroups. It contains 1427 samples and 4322 dimensions. The *ringnorm-DELVE*, which is an implementation of Leo Breiman’s ringnorm example, is a 20 dimensional, 2 class classification with 7400 samples^54^. Another example, *Madelon*, introduced an artificially generated dataset 2600 samples and 500 dimensions, where only 5 principal and 20 derived variables containing information. Instead, we used a much more complicated example than *Madelon*, an NIHS dataset^50^ that we used to test *DeepMapper* in the first place. We attempted to run *DeepInsight* with NIHS data, but we could not get it to train properly and for this reason we cannot supply a comparison.

The most complex problem published by *DeepInsight* was the analysis of a public RNA sequencing gene expression dataset from TCGA (https://cancergenome.nih.gov/) containing 6216 samples of 60,483 genes or dimensions, of which *DeepInsight* used 19,319. We selected this example as the second demonstration of application of *DeepMapper* to high dimensional data, as well as a benchmark for comparison with *DeepInsight*.

**Figure 6:**
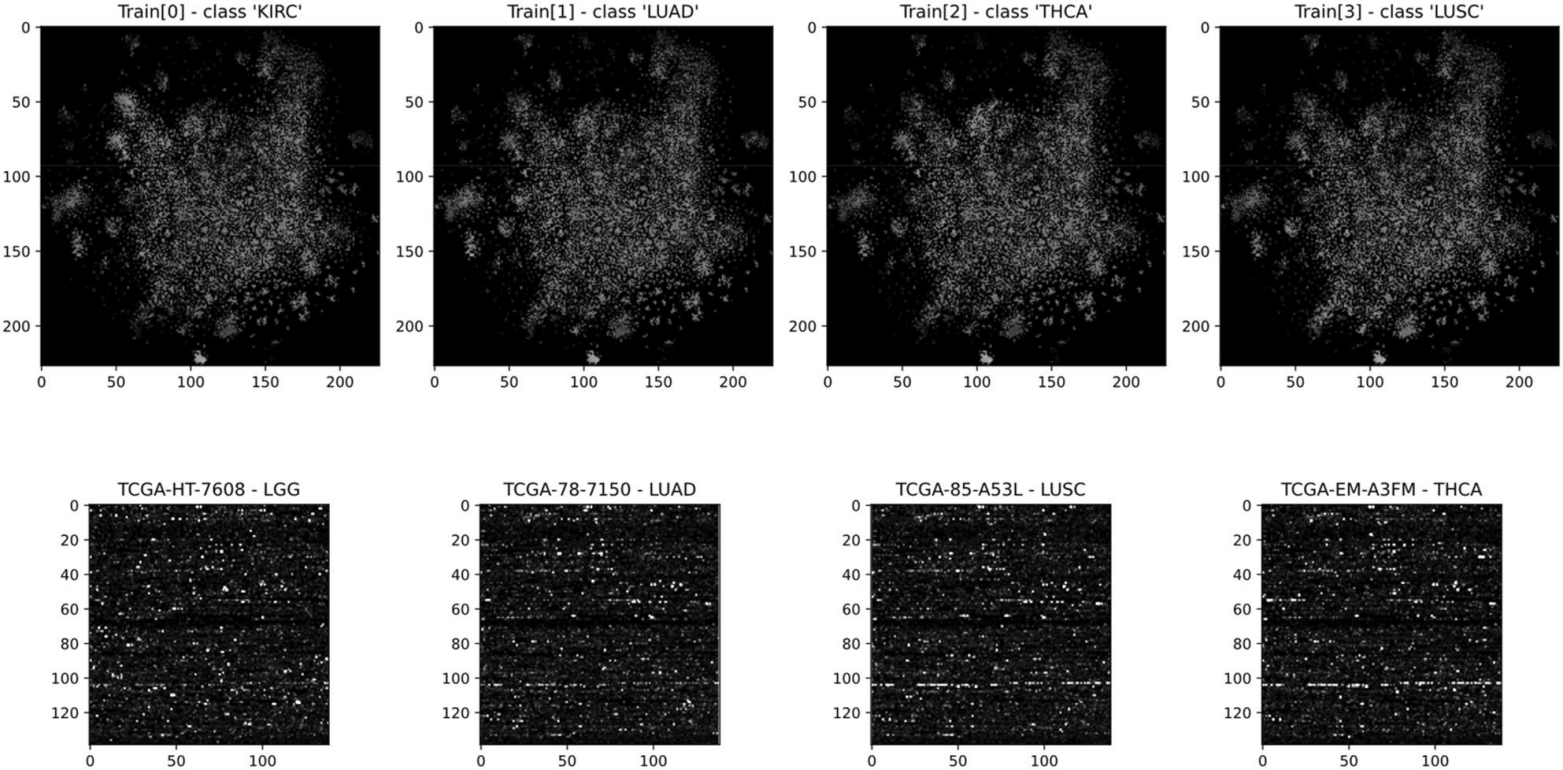
Analysis of TCGA data by *DeepInsight* vs *DeepMapper:* The image on the top was generated by *DeepInsight* using its default values and a t-SNE transformer supplied by *DeepInsight*. The image at the bottom was generated by *DeepMapper.* Image conversion and training speeds and the analysis results can be found in Table 2.

We generated the data using the R script offered by *DeepInsight*^54^ and ran *DeepMapper* as well as *DeepInsight* using the generated dataset to compare accuracy and speed. In this test *DeepMapper* exhibited much improved processing speed with near identical accuracy (Table 2, Fig. 6).

**Table 2:** DeepInsight-*DeepMapper* comparison. (*Note: DeepInsight uses 224*224 image resolution, but this resolution is only required if a pre-trained network is utilized).

	DeepInsight	DeepMapper
Image conversion time	I min 34 s	334 ms
Image size	224 × 224*	139 × 139
Training time	23 min 37 s	8 min 56 s
Accuracy	Train: 1.00 Test: 0.976	Train: 1.00 Test: 0.975

## Discussion

CNNs are fundamentally sophisticated pattern matchers that can establish intricate mappings between input features and output representations^6^. They excel at transforming various inputs into outputs, including identifying classes or bounding boxes, through a series of operations involving convolution, pooling, and activation functions^7,56^.

Even though CNNs are in the centre of many of today’s revolutionary AI systems from self-driving cars to generative AI systems such as *Dall-E-2*, *MidJourney* and *Stable Diffusion*, they are still not well understood nor efficiently utilised, and their usage beyond image analysis has been limited.

While CNNs used in image analysis are constrained historically and practically to a 224 × 224 matrix or a similar fixed size input, this limitation arises for pre-trained models. When CNNs have not been pre-trained, one can select a much wider variety of sizes as input shape depending on the CNN architecture. Some CNNs are more flexible in their input size that implemented with adaptive pooling layers such as ResNet18 using adaptive pooling^57^. This provides flexibility to choose optimal sizes for the task in hand for non-image applications, as most non-image applications will not use pre-trained CNNs.

Here we have demonstrated uses of CNNs that are outside the norm. There is a need for analysis of complex data with many thousands of features that are not primarily images. There is also a lack of tools that offer minimal conversion of non-image data to image-like formats that then can easily be processed with CNNs in classification and clustering tasks. As a lot of this data is coming from complex systems that have a lot of features, *DeepMapper* offers a way of investigating such data in ways that may not be possible with traditional approaches.

Although *DeepMapper* currently uses CNN as its AI component, alternative analytic strategies can easily be substituted in lieu of CNN with minimal changes, such as *Vision Transformers*^18^ or *RetNets*^21^, which have great potential for this application. While *Transformers* and *RetNets* have input size limitations for inference in terms of number of tokens. *Vision Transformers* can handle much larger inputs by dividing images to segments that incorporate multiple pixels^18^. This type of approach would be applicable to both *Transformers* and *RetNets*, and future architectures. *DeepMapping* can leverage these newer architectures, and others, in the future^57^.

## Data Availability

DeepMapper is released as an open source tool on GitHub https://github.com/tansel/deepmapper. Data that is not available from GitHub because of size constraints can be requested from the authors.
